# Age-stratified analysis of HTO and UKA clinical effects in cross-indicated anterior medial osteoarthritis

**DOI:** 10.1007/s00590-024-03944-4

**Published:** 2024-04-17

**Authors:** Han Xu, Huali Tu, Tianzuo Zhao, Daofei Xu, Qinglong Yu, Long Liao, Tao Zhang, Bo Shi

**Affiliations:** Mianyang Central Hospital, School of Medicine, University of Electronic Science and Technology of China, No. 12, Changjia Lane, Jingzhong Street, Mianyang, 621000 China

**Keywords:** Anterior medial osteoarthritis, High tibial osteotomy, Unicompartmental knee arthroplasty, Clinical effects

## Abstract

**Purpose:**

To compare clinical outcomes of high tibial osteotomy (HTO) and unicompartmental knee arthroplasty (UKA) for anterior medial osteoarthritis (AMOA) as well as offer surgical recommendations through age stratification.

**Methods:**

Between May 2019 and May 2021, 68 cross-indicated AMOA patients were analyzed. The patients were divided into HTO and UKA groups and further into two age groups of 55–60 and 60–65 years. Additionally, general data, visual analog scale (VAS) score, and Hospital for Special Surgery knee score (HSS) were analyzed.

**Results:**

All the patients were followed up for 18 months. Knee joint HSS significantly improved, and VAS score decreased in both groups (*P* < 0.05). In the 55–60 age group, HTO showed superior knee HSS at 1 and 3 months (*P* < 0.05), with no significant difference at 6, 12, and 18 months. HTO had a significantly lower VAS score at one month, and the VAS scores of the two groups decreased gradually with no significant difference. In the 60–65 age group, the UKA group showed superior knee joint HSS at one month, with no significant difference at 3, 6, 12, and 18 months. The UKA group had a significantly lower VAS score at one month, and both groups’ VAS scores decreased gradually with no significant difference.

**Conclusion:**

Both methods yield satisfactory results for AMOA cross-indications, improving knee joint function. The observed recovery trends have implications for personalized surgical recommendations, guiding interventions based on age-specific considerations for optimal outcomes in anterior medial osteoarthritis cases.

## Introduction

With a growing understanding of the evolution of knee osteoarthritis (KOA) and the widespread adoption of the concept of "knee protection" both domestically and internationally, high tibial osteotomy (HTO) and unicompartmental knee arthroplasty (UKA) have emerged as primary treatment modalities for patients in the mid to late stages of KOA, particularly those with anterior medial osteoarthritis (AMOA) of the knee [1, 2].

Despite variations in the treatment principles between HTO and UKA, there are overlapping surgical indications. While studies have reported the clinical efficacy of HTO and UKA in the treatment of AMOA, a consensus on the preferred surgical approach for this subgroup of patients with cross-indications is yet to be reached [3].

To address this gap, this article presents a case–control study conducted on AMOA patients with cross-indications who underwent either HTO or UKA at our hospital. The study compares the clinical efficacy of these two patient groups, considering variations across different age groups. The aim is to offer improved recommendations for physicians and patients when making surgical selections.

## Methods

### Inclusion and exclusion criteria

Inclusion criteria: Intersection of indications for HTO and UKA: (1) Patients who meet the diagnosis of knee joint AMOA and have a Kellgren–Lawrence grade of III; (2) patients ranging in age from 55 to 65 years; (3) preoperative MRI of the affected knee indicates intact medial and lateral collateral ligaments and cruciate ligaments of the knee joint; and (4) patients with preoperative proximal medial tibial deformity of the affected limb ranging from 5 to 10°.

Exclusion criteria: (1) Patients with a body mass index (BMI) > 35 kg/m^2^; (2) patients with multi-compartment wear of the knee joint; (3) individuals with other functional abnormalities such as knee joint ligaments; and (4) patients with non-degenerative knee osteoarthritis such as traumatic and rheumatoid arthritis.

### General information

A total of 68 patients with cross-indications of AMOA, admitted to our hospital between May 2019 and May 2021, were included in this study. Patients were categorized into two groups based on the surgical method chosen: HTO (36 cases) and UKA (32 cases). In the HTO group, there were 22 males and 14 females with an average age of 58.66 ± 2.74 years; 16 cases involved the left knee and 20 involved the right knee. The BMI averaged 24.77 ± 3.05 kg/m^2^, with preoperative HSS scores at 56.67 ± 4.29 points and preoperative VAS scores at 4.15 ± 0.91 points. The UKA group comprised 14 males and 18 females with an average age of 59.50 ± 3.70 years old; 14 cases involved the left knee and 18 cases the right knee. The BMI averaged 23.76 ± 3.08 kg/m^2^, with preoperative HSS scores at 55.94 ± 4.70 points and preoperative VAS scores at 3.84 ± 0.85 points. No significant differences were observed between the two groups concerning sex (*x*^*2*^ = 0.271, *P* = 0.602), age (*t* =  − 0.985, *P* = 0.332), affected side (*x*^*2*^ = 0.617, *P* = 0.432), BMI (*t* = 1.273, *P* = 0.208), or preoperative HSS and VAS scores of the knee joint (*t* = 0.478, *P* = 0.636; *t* = 1.611, *P* = 0.119).

### Operative technique

Both patient groups underwent standardized surgical procedures under general anesthesia, performed by the same chief surgeon and team. The HTO group underwent open-wedge high tibial osteotomy utilizing a Tomofix high medial tibial plate (DePuy Synthes, USA). Patients in the UKA group were treated with a mobile-bearing Oxford Unicondylar Prosthesis System (Zimmer Biomet, USA).

### Perioperative management

Both groups followed an identical perioperative accelerated rehabilitation process. This involved the administration of tranexamic acid and cefuroxime 30 min before surgery to minimize bleeding and prevent infection. Standard postoperative care included low molecular weight heparin anticoagulation and conventional analgesics. Upon returning to the ward, guidance on ankle pump exercise and quadriceps muscle contraction training commenced. Postoperative day one initiated knee joint flexion, extension, and straight leg elevation exercises. On the second day post-surgery, lower limb vascular ultrasound and radiographic re-examination were performed. In the absence of abnormalities, active functional exercises with assistive devices were encouraged.

### Observation index

General information (sex, age, affected side, and BMI) of the two patient groups along with preoperative and postoperative VAS scores and knee HSS scores at 1, 3, 6, 12, and 18 months was recorded.

### Statistical analysis

Data analysis utilized SPSS software (version 22.0; IBM, Armonk, NY, USA). Quantitative data were represented as mean ± standard deviation. A t test was used for comparison, while count data were illustrated as an example, and the *x*^*2*^ test was used for intergroup comparisons. Differences were considered statistically significant at *P* < 0.05.

## Results

All patients underwent an 18-month follow-up. HSS scores significantly improved, and VAS score markedly decreased in both groups before surgery, after surgery, and one month post-surgery, with statistical significance (*P* < 0.05) (Table 1). The patients were further divided according to their ages into two groups: 55–60 years and 60–65 years (Tables 2 and 3).

**Table 1:** Comparison of HSS and VAS scores of two groups of knee joints before and after surgery

	HSS		VAS	
	HTO	UKA	HTO	UKA
Preop	56.67 ± 4.29	55.94 ± 4.70	4.15 ± 0.91	3.84 ± 0.85
1 month postop	75.63 ± 2.54	79.38 ± 2.83	2.48 ± 0.51	1.93 ± 0.95
*t* Value	− 18.817	− 23.160	7.806	9.649
*P* value	0.000	0.000	0.000	0.000

**Table 2:** Patient demographics (55–60 years old)

Groups	*N*	Age (years)	Sex (male/female)	BMI (kg/m^2^)	Surgical side (left/right)	HSS score	VAS scores
HTO	19	56.47 ± 1.54	10 9	24.13 ± 3.48	8 11	56.74 ± 4.71	4.32 ± 0.89
UKA	19	56.79 ± 1.47	10 9	24.39 ± 3.24	9 10	56.37 ± 4.03	4.21 ± 0.79
*t x*^*2*^ value		1.064	1.191	− 1.453	0.787	1.278	0.369
*P* value		0.301	0.235	0.149	0.435	0.217	0.716

**Table 3:** Patient demographics (60–65 years old)

Groups	*N*	Age (years)	Sex (male/female)	BMI (kg/m^2^)	Surgical side (left/right)	HSS score	VAS scores
HTO	17	62.08 ± 1.19	9 8	26.01 ± 2.33	9 8	55.46 ± 4.33	4.15 ± 0.90
UKA	13	62.77 ± 1.79	6 7	25.41 ± 2.12	6 7	54.77 ± 4.60	3.92 ± 0.86
*t/x*^*2*^ value		− 1.511	2.356	2.341	− 2.124	0.512	0.601
*P* value		0.157	0.811	0.134	1.322	0.618	0.553

In the 55–60 years age range, the HSS score of the knee joint in the HTO group was 79.05 ± 3.01 points one month post-surgery and 83.53 ± 4.02 points 3 months post-surgery. The knee joint HSS score of the UKA group was 75.68 ± 2.60 points one month and 81.11 ± 3.75 points 3 months post-surgery. The HSS of the HTO group was significantly better than that of the UKA group at 1 and 3 months post-surgery (*P* < 0.05). At 6, 12, and 18 months post-surgery, the HSS of both groups gradually improved; however, there was no statistically significant difference between them (*P* > 0.05) (Table 4). The VAS score of the HTO group was 1.89 ± 0.94 points one month after surgery, whereas the VAS score of the UKA group was 2.47 ± 0.51 points. The VAS score of the HTO group was significantly lower than that of the UKA group one month post-surgery (*P* < 0.05). Subsequently, at 3, 6, 12, and 18 months postoperatively, the VAS scores of both groups gradually decreased; however, no statistically significant difference was observed between the two groups (*P* > 0.05) (Table 5).

**Table 4:** Comparison of knee joint HSS score between the two groups (55–60 years old)

Groups	1 month postop	3 months postop	6 months postop	12 months postop	18 months postop
HTO	79.05 ± 3.01	83.53 ± 4.02	90.16 ± 2.72	92.95 ± 2.25	93.53 ± 3.13
UKA	75.68 ± 2.60	81.11 ± 3.75	88.89 ± 2.13	91.16 ± 3.10	92.84 ± 2.69
*t* value	3.427	1.861	3.274	1.797	0.770
*P* value	0.003	0.004	0.079	0.089	0.451

**Table 5:** Comparison of VAS scores between the two groups (55–60 years old)

Groups	1 month postop	3 months postop	6 months postop	12 months postop	18 months postop
HTO	1.89 ± 0.94	1.00 ± 0.88	1.18 ± 0.83	0.47 ± 0.51	0.48 ± 0.52
UKA	2.47 ± 0.51	1.16 ± 0.90	1.26 ± 0.80	0.57 ± 0.50	0.42 ± 0.51
*t* value	− 2.157	− 0.497	− 0.462	− 0.697	0.294
*P* value	0.045	0.625	0.650	0.494	0.772

In the 60–65 years age range, the HSS score of the knee joint in the HTO group was 75.77 ± 2.59 points one month post-surgery, while the knee joint HSS score of the UKA group was 79.85 ± 2.57 points at the same interval. The knee joint HSS of the UKA group significantly surpassed that of the HTO group one month post-surgery (*P* < 0.05). Subsequent assessments at 3, 6, 12, and 18 months demonstrated gradually improvements in the HSS scores of knee joints in both groups; however, no statistically significant difference existed between the two groups (*P* > 0.05) (Table 6). The VAS score of the HTO group was 2.62 ± 0.51 points one month after surgery, while the VAS score of the UKA group was 2.00 ± 1.00 at the same interval. The VAS score of the UKA group was significantly lower than that of the HTO group one month after surgery, indicating a statistically significant difference (*P* < 0.05). Follow-up assessments at 3, 6, 12, and 18 months postoperatively revealed a gradual decrease in VAS scores for both groups, with no statistically significant difference between them (*P* > 0.07) (Table 7).

**Table 6:** Comparison of knee joint HSS scores between the two groups (60–65 years old)

Groups	1 month postop	3 months postop	6 months postop	12 months postop	18 months postop
HTO	75.77 ± 2.59	81.38 ± 3.88	91.69 ± 2.36	91.92 ± 2.33	94.85 ± 1.86
UKA	79.85 ± 2.57	83.15 ± 3.82	80.77 ± 1.88	91.00 ± 3.30	93.77 ± 2.95
*t* value	− 4.709	− 1.402	0.993	1.251	1.117
*P* value	0.001	0.186	0.341	0.235	0.286

**Table 7:** Comparison of VAS scores between the two groups (60–65 years old)

Groups	1 month postop	3 months postop	6 months postop	12 months postop	18 months postop
HTO	2.62 ± 0.51	1.15 ± 0.89	1.08 ± 0.76	0.69 ± 0.48	0.53 ± 0.52
UKA	2.00 ± 1.00	0.92 ± 0.86	0.77 ± 0.83	0.54 ± 0.52	0.46 ± 0.51
*t* value	2.551	0.640	1.000	0.805	0.365
*P* value	0.025	0.534	0.337	0.436	0.721

**Fig. 1:**
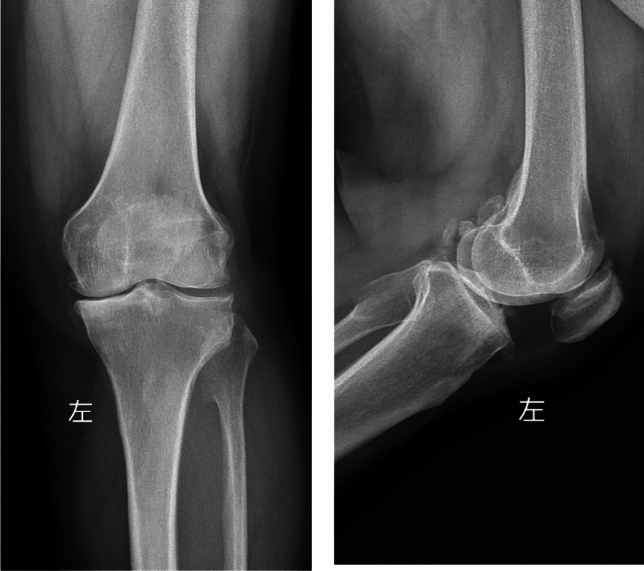
Anterior and lateral X-ray films of the knee joint before operation showing changes in osteoarthritis of the left knee joint, especially in the medial compartment

**Fig. 2:**
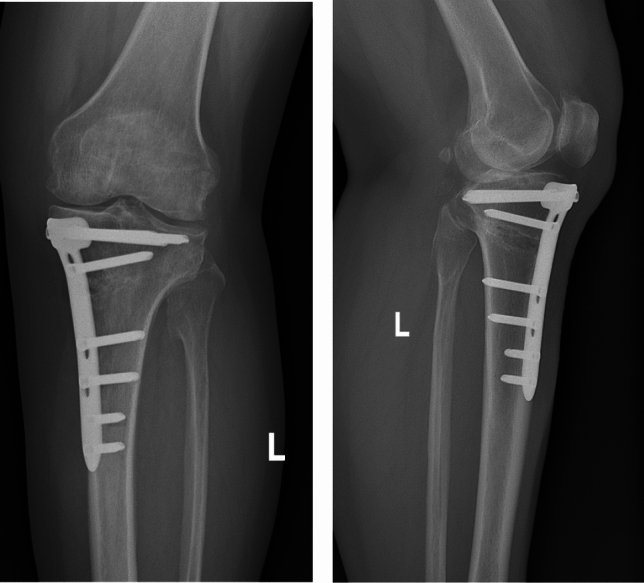
Positive and lateral X-ray films of the knee joint showing changes after high osteotomy and internal fixation of the left tibia, and the osteotomy surface and internal fixation position were satisfactory

A typical case of HTO involved a 58-year-old female patient with left knee joint pain, who was admitted for 4 years. After admission, a comprehensive evaluation, including the patient's medical history, specialized physical examination, and preoperative radiography, led to the diagnosis of left knee osteoarthritis (Kellgren–Lawrence grade III). Under general anesthesia, a left open-wedge HTO was successfully performed, resulting in good postoperative recovery. Imaging data before and after surgery are shown in Figs. 1 and 2, respectively.

**Fig. 3:**
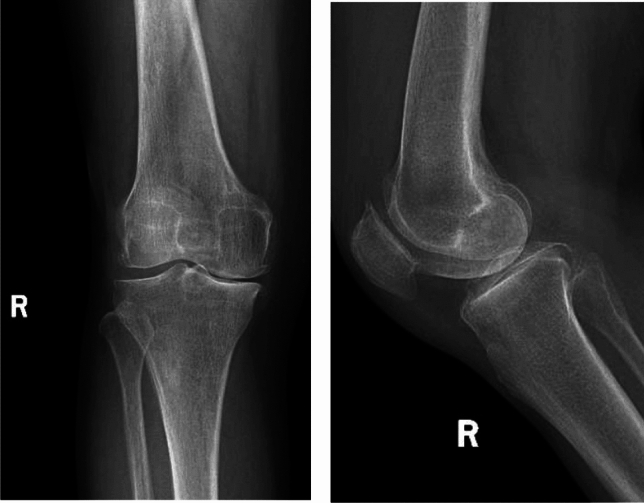
Anterior and lateral radiography films of the knee joint before operation showing the changes in osteoarthritis of the right knee joint, especially in the medial compartment

**Fig. 4:**
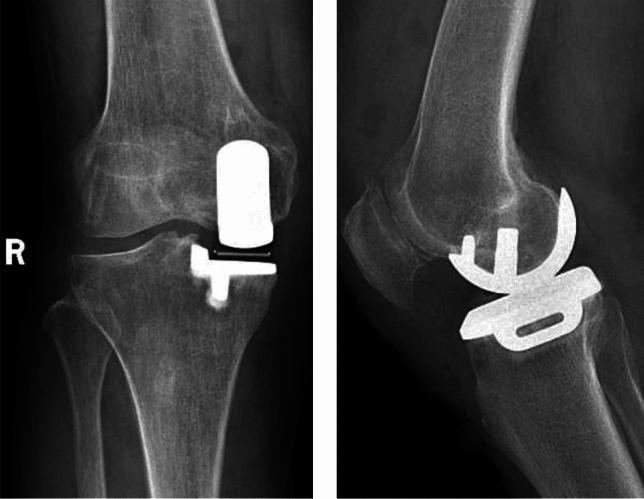
Radiographs of the anterior and lateral position of the knee joint showing changes after single condylar replacement of the right knee joint. The position of the prosthesis is satisfactory

A typical case of UKA involved a 62-year-old female patient with right knee joint pain who was admitted for 3 years. Post-admission, a thorough assessment of the patient's medical history, specialized physical examination, and preoperative radiography confirmed the diagnosis of right knee osteoarthritis (Kellgren–Lawrence grade III). Under general anesthesia, a single-condyle replacement surgery of the right knee joint was performed, leading to a favorable postoperative recovery. Imaging data from before and after surgery are shown in Figs. 3 and 4, respectively.

## Discussion

Continuous and extensive research on knee osteoarthritis has been conducted globally, along with promotion of knee protection concepts and tiered treatment of knee osteoarthritis. HTO and UKA, recognized as the two most effective surgical methods for treating knee joint AMOA, have garnered increased attention and extensive research from orthopedic professionals [4, 5]. Traditional indications for HTO include single-compartment knee osteoarthritis, age < 65 years, absence of knee instability, knee flexion deformity < 10°, and tibial varus deformity > 5° [6]. Traditional indications for UKA comprise knee joint single-compartment osteoarthritis, age > 55 years, good knee joint mobility, absence of ligament damage, and tibial varus deformity < 10° [7]. Despite the strict indications for both HTO and UKA, and favorable prognoses achieved under these criteria and standard surgical procedures, there is an intersection in their indications. The treatment methods for this specific patient subgroup are subject to controversy. Therefore, we collected clinical data from this patient cohort in our hospital to gain deeper insights into the clinical efficacy of HTO and UKA in cases of cross-indications through comparative studies.

Similar to most research findings [8], HTO and UKA can yield favorable clinical outcomes in patients with knee osteoarthritis, especially AMOA. Our study observed significant improvement in knee HSS and VAS pain scores for one month after surgery in patients who underwent either procedure. Our investigation revealed that knee joint HSS scores of patients aged 55–60 years in the HTO group were significantly superior to those in the UKA group 1 and 3 months postoperatively, with their VAS scores at one month also significantly lower than the UKA group. Conversely, patients in the 60–65-year-old UKA age group exhibited significantly better knee HSS and VAS scores than the HTO group one month after surgery. Younger patients undergoing HTO demonstrated faster recovery compared to those who undergoing UKA, while older patients undergoing UKA exhibited quicker recovery than those undergoing HTO. Our findings suggest that HTO, involving osteotomy correction without intra-articular surgery, preserves proprioceptive sensation, leading to faster recovery of knee joint function and pain sensation in younger patients compared to the intra-articular surgical method of UKA. However, in older patients, slower healing of the HTO osteotomy surface creates a significant impediment to postoperative recovery, resulting in a slower early recovery compared to UKA. This aligns with the findings of Nerhus et al. [9], indicating that the absence of bone grafting or other procedures in the HTO gap may affect knee joint function recovery, prolonging the time required to reach or surpass pre-surgery levels of physical activity. While postoperative recovery speed is influenced by various factors, our conclusion finds support in our research results and numerous domestic and foreign research reports [10]. Bouguennec et al. [11] conducted a multicenter retrospective study comparing 488 HTO patients and 284 UKA patients, reporting that, although multifactorial, both HTO and UKA yielded positive functional outcomes for patients aged 55–65. Ijka et al. [12] conducted a retrospective study with 123 HTO and 118 UKA patients, revealing higher satisfaction among elderly UKA patients compared to younger HTO patients. Similarly, Shen et al. [13] observed a tendency for HTO to be favorable for younger and more active patients, whereas UKA was deemed more suitable for older and less active patients. Walker et al. [14] observed a significant improvement in knee joint function scores for patients aged < 60 years who underwent UKA over an average follow-up period of 53 months. However, several studies have confirmed that HTO is more cost-effective and effective than UKA in patients aged < 60 years [15–17].

Both HTO and UKA aim to improve symptoms such as knee pain and limited mobility, delay further progression of osteoarthritis, and consequently, postpone or eliminate the need for total knee replacement surgery. Therefore, the postoperative survival rate serves as a crucial prognostic indicator. In a 10-year follow-up, Song et al. [18] found no significant difference in survival rates between the two groups: HTO exhibited a 91% rate and UKA showed 87.1%. Similarly, Bouguennec et al. [11] reported a 10-year survival rate of 74.3% for HTO and 71% for UKA, with no significant difference observed. Unfortunately, our follow-up period of at least 18 months might be insufficient to draw definitive conclusions regarding long-term outcomes, including survival rates. Furthermore, our retrospective approach and relatively small sample size may have affected the results.

In conclusion, both HTO and UKA demonstrate satisfactory therapeutic effects in patients with AMOA cross-indications, significantly enhancing knee joint function. In the age range of 55–60 years, HTO patients experience quicker recovery than UKA patients; for individuals between 60 and 65 years, UKA patients exhibit faster recovery than HTO patients. Therefore, under normal circumstances, HTO is recommended for younger patients with AMOA cross-indications, while older patients are advised to opt for UKA. Further research could explore the long-term outcomes, including survival rates, in a larger and more diverse patient population to enhance the generalizability of findings and inform more comprehensive clinical recommendations.

## Data Availability

Not applicable.
